# Pathogenetic Insights into Developmental Coordination Disorder Reveal Substantial Overlap with Movement Disorders

**DOI:** 10.3390/brainsci13121625

**Published:** 2023-11-23

**Authors:** Martinica Garofalo, Fleur Vansenne, Deborah A. Sival, Dineke S. Verbeek

**Affiliations:** 1 Department of Pediatric Neurology, Beatrix Children’s Hospital, University Medical Center Groningen, University of Groningen, 9713 GZ Groningen, The Netherlands; m.garofalo@umcg.nl (M.G.); d.a.sival@umcg.nl (D.A.S.); 2 Expertise Center Movement Disorders Groningen, University Medical Center Groningen (UMCG), 9713 GZ Groningen, The Netherlands; f.vansenne@umcg.nl; 3 Department of Genetics, University Medical Center Groningen, University of Groningen, 9713 GZ Groningen, The Netherlands

**Keywords:** Developmental Coordination Disorder (DCD), genetics, movement disorders, pathogenetic spectrum

## Abstract

Developmental Coordination Disorder (DCD) is a neurodevelopmental condition characterized by non-progressive central motor impairments. Mild movement disorder features have been observed in DCD. Until now, the etiology of DCD has been unclear. Recent studies suggested a genetic substrate in some patients with DCD, but comprehensive knowledge about associated genes and underlying pathogenetic mechanisms is still lacking. In this study, we first identified genes described in the literature in patients with a diagnosis of DCD according to the official diagnostic criteria. Second, we exposed the underlying pathogenetic mechanisms of DCD, by investigating tissue- and temporal gene expression patterns and brain-specific biological mechanisms. Third, we explored putative shared pathogenetic mechanisms between DCD and frequent movement disorders with a known genetic component, including ataxia, chorea, dystonia, and myoclonus. We identified 12 genes associated with DCD in the literature, which are ubiquitously expressed in the central nervous system throughout brain development. These genes are involved in cellular processes, neural signaling, and nervous system development. There was a remarkable overlap (62%) in pathogenetic mechanisms between DCD-associated genes and genes linked with movement disorders. Our findings suggest that some patients might have a genetic etiology of DCD, which could be considered part of a pathogenetic movement disorder spectrum.

## 1. Introduction

Developmental Coordination Disorder (DCD) is one of the most common neurodevelopmental disorders [1], affecting about 5% of children over the age of 5 years [2,3] and often persisting into adulthood (in 30–70% of cases) [4]. The symptoms manifest as non-progressive central motor impairments, including motor apraxia, clumsiness, impaired limb coordination, and gait instability [2,3,5]. Frequently accompanying non-motor deficits are poor executive functioning, attention deficit/hyperactivity disorder (ADHD), autism-spectrum disorder (ASD), and specific learning disorder, including specific language impairment, developmental dyslexia, and reading disorder, among other learning disabilities [2,3,6,7]. DCD can be diagnosed when all the following diagnostic criteria are met: (a) the acquisition of motor skills is delayed for the child’s age; (b) the symptoms start early in the development and significantly affect the child’s daily activities; (c) neurological disorders that could be explanatory for the phenotype have been excluded, including movement disorders, hypotonia, muscle weakness, visual impairment, and moderate to severe intellectual disability (ID) [2,3,5]. The clinical identification of DCD is often challenging [8,9,10], as mild features of movement disorders, including ataxia, dystonia and/or chorea, could be observed [6,11], and the motor phenotype of DCD may resemble physiologically immature motor features of young, typically developing children [3,12].

Despite the identification of putative risk factors for DCD, including male sex, prematurity, and perinatal oxygen perfusion problems [2,13,14,15], the underlying etiology remains unknown. Recently, the hypothesis of a genetic substrate of DCD was suggested by studies on family aggregation [16] and heritability of DCD, estimated to ≥70% in monozygotic twins [17,18]. This genetic hypothesis is further supported by the high prevalence of comorbid neurodevelopmental disorders (NDDs) in DCD, including ADHD (about 50%) and ASD (about 47%) [3,19,20,21]. In a recent copy-number variation (CNV) analysis in patients with DCD, rare CNVs in genomic loci were identified, encompassing genes previously associated with ADHD and ASD, among other NDDs [22]. These findings suggest a shared genetic etiology between DCD and its comorbid NDDs [15,19,23]. So far, two genome-wide association studies performed in children with phenotypes resembling DCD have failed to identify major risk genes for DCD [24,25]. In mice with DCD-like phenotypic traits, a recent quantitative trait locus analysis identified candidate genes correlating with impaired murine gait and coordination [26]. Until now, these findings have not been replicated in patients with DCD [26]. Therefore, despite the strong indications for genetic underpinnings, comprehensive knowledge about the associated genes and underlying pathogenetic mechanisms of DCD is still lacking.

Based on the current evidence, in the present study we hypothesized a genetic substrate in a subgroup of patients with DCD. To further investigate this, we first aimed at identifying all genes reported in the literature in patients with a diagnosis of DCD according to the official diagnostic guidelines [2,3]. Second, we aimed at exposing the underlying temporal and tissue gene expression patterns and brain-specific biological mechanisms of DCD-associated genes. Moreover, due to the possible clinical presence of mild movement disorder features in DCD, we hypothesized that DCD and movement disorders may share similar pathogenetic mechanisms. Accordingly, our third aim was to explore putative shared pathogenetic pathways between DCD and the most frequent pediatric movement disorders with a known genetic component, including ataxia, chorea, dystonia, and myoclonus [27,28].

## 2. Materials and Methods

### 2.1. First Aim: Gene Identification in the Literature

#### Comprehensive Literature Review and Gene Inclusion Criteria

The first aim of our study was to identify all genes associated with DCD in the literature, either described with single nucleotide polymorphisms (SNPs) or residing within loci where CNVs were reported. For this, we performed a comprehensive literature search using PubMed of the National Center for Biotechnology Information (Bethesda, MD, USA: National Library of Medicine, US. Available online: https://pubmed.ncbi.nlm.nih.gov; accessed on 17 May 2022) (Supplementary File S1). Prior to gene selection, we defined the following inclusion criteria, according to the official guidelines [2,3]. A gene was selected for inclusion when all of the following conditions were met: (a) there was a clinical diagnosis of DCD (according to the DSM-4 or DSM-5 criteria); (b) genetic variants (SNPs/CNVs) were reported in studies using human genetic material; and (c) the gene was not primarily associated with neurological conditions that are phenotypically explanatory for the coordination impairments, such as movement disorders, moderate to severe ID (defined in the ICD-10 as an IQ of <50 [29]), or neuromuscular, visual or vestibular disorders [3]. To verify whether genes were primarily associated with the abovementioned neurological conditions, we consulted PubMed and Online Mendelian Inheritance in Man (OMIM; McKusick-Nathans Institute of Genetic Medicine, Johns Hopkins University. Baltimore, MD. Available online: https://omim.org; accessed on 21 May 2022).

**Figure 1:**
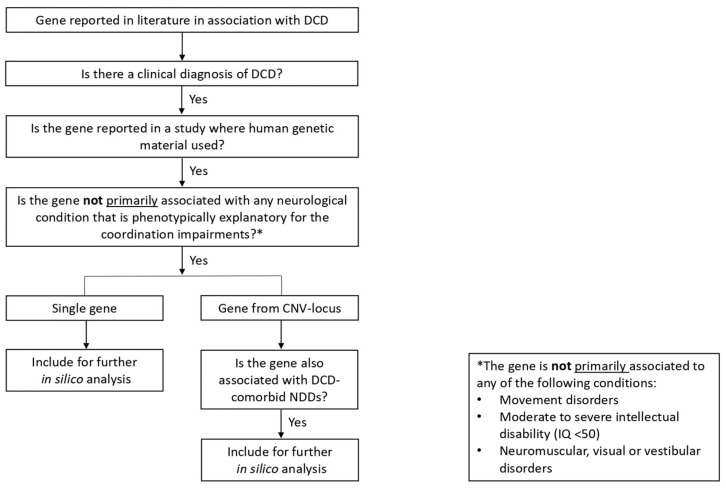
Gene inclusion criteria for the present study. Flowchart depicting the process of gene inclusion based on the inclusion criteria that we defined according to the official guidelines [2,3]. First, we examined whether there was a clinical diagnosis of DCD according to the DSM-4 or DSM-5 criteria. We only included genes reported in patients with a clinical diagnosis of DCD. Genes reported in patients who were suspected of DCD or had a similar phenotype but no clinical diagnosis, were not included. Second, we screened for genetic variants that were reported in studies using human genetic material. Candidate genes from mice studies were therefore not included. Genetic variants were reported as either single nucleotide polymorphism (SNP) or copy-number variations (CNV) in a genetic locus. Third, we investigated the disease associations of these genes. According to the official diagnostic criteria, we only included genes that were not primarily associated with any of the following conditions: movement disorders, moderate to severe ID (defined in the ICD-10 as an IQ of <50), or neuromuscular, visual or vestibular disorders. If all of the abovementioned criteria were met, then we included the gene for further in silico analysis. For genes residing within a CNV-locus, we further checked whether the gene was previously associated with DCD-comorbid neurodevelopmental disorders (NDDs), such as attention deficit/hyperactivity disorder or autism-spectrum disorder. If that was the case, then the gene was included for further in silico analysis. DCD = Developmental Coordination Disorder; SNP = single nucleotide polymorphism; CNV = copy-number variation; NDD = neurodevelopmental disorder.

Moreover, for genes within a genetic locus where CNVs were associated with DCD in the literature, the additional inclusion criterion of being associated with DCD-comorbid NDDs, such as ADHD or ASD, had to be met (Figure 1).

We retrieved genes from CNV-loci which were not reported in the original manuscripts using the exact cytogenetic coordinates in UCSC Genome Browser (Human Genome Browser, GRCh37/hg19; 2009. Available online: http://genome.ucsc.edu; accessed on 22 May 2022) [30]. For all CNVs described in the literature, the reference genome build was GRCh37/hg19.

### 2.2. Second Aim: Analysis of Pathogenetic Mechanisms Underlying DCD

#### 2.2.1. Temporal Gene Expression Analysis

As part of the second aim of our study, we first investigated the temporal expression patterns of the genes associated with DCD. According to the diagnostic criterion C of the DSM-5, the onset of DCD symptoms occurs in the early developmental period [2]. Using publicly accessible reads per kilobase per million (RPKM) RNAseq data from BrainSpan (Gencode v10, Atlas of the Developing Human Brain, https://www.brainspan.org/static/home; accessed on 24 May 2022), we explored whether the expression of genes associated with DCD was regulated during the development of the cerebellum, basal ganglia, and frontal cortex. These structures are the main anatomical regions involved in planning, coordination, and motor control [31,32], which are typically impaired features in DCD [2,3]. We reclassified 23 developmental stages available in BrainSpan into 7 phases (S1–S7, from 8 postconceptional weeks until the age of 19 years [33]; Supplementary Figure S1), as previously described [34,35].

#### 2.2.2. Tissue Gene Expression Analysis

Second, we explored the tissue expression patterns of the genes associated with DCD. Because DCD is a central cause of motor incoordination [36], we investigated whether the genes associated with DCD were specifically expressed in any central nervous system (CNS) structure, using the multi-gene query function of Genotype-Tissue Expression (GTEx) Portal (Analysis Release V8, available at: https://www.gtexportal.org; accessed on 24 May 2022) [37]. The available CNS structures included the amygdala, anterior cingulate cortex, caudate nucleus, cerebellum, frontal cortex, hippocampus, hypothalamus, nucleus accumbens, putamen, spinal cord, and substantia nigra. Publicly accessible RNAseq data were available as Transcript per Million (TPM).

#### 2.2.3. Functional Enrichment and Biological Pathway Analysis in the DCD-Associated Gene Co-Expression Network

Third, we investigated the biological mechanisms underlying the genes associated with DCD. For this, we performed functional enrichment using MetaBrain (https://network.metabrain.nl; accessed on 30 May 2022) [38]. The resulting brain-specific DCD-associated gene co-expression network was procedurally enriched with 200 genes predicted to be functionally similar to the DCD-associated genes (hereafter referred as DCD-predicted genes; Supplementary File S2). Then, we performed biological pathway analysis using Metascape (version 3.5, available at: http://metascape.org; accessed on 30 May 2022) [39], where similar biological pathways are clustered together, as described elsewhere [35,39]. Afterwards, we used gProfiler (database version: Ensembl 104, Ensembl Genomes 51, Wombase ParaSite 15. Available online: https://biit.cs.ut.ee/gprofiler/gost; accessed on 30 May 2022) [40], ToppGene Suite (https://toppgene.cchmc.org/enrichment.jsp; accessed on 30 May 2022) [41] and MetaBrain to verify the reproducibility of these data. Throughout all analyses, we annotated the gene ontology (GO, http://geneontology.org/; accessed on 30 May 2022) [42], REACTOME (https://reactome.org/; accessed on 30 May 2022) [43], WikiPathways (https://www.wikipathways.org/; accessed on 30 May 2022) [44] and KEGG (https://www.kegg.jp/kegg/; accessed on 30 May 2022) [45] biological pathways with a statistically significant adjusted *p*-value of ≤10^−5^, corrected for multiple testing (Bonferroni). Clustered data were visualized through Cytoscape 3.9.1 (https://cytoscape.org; accessed on 1 June 2022) [46].

We also investigated whether there was an overlap between the 200 DCD-predicted genes in the DCD-associated gene co-expression network (Supplementary File S2) and the genes from CNV-loci associated with DCD in the literature. The presence of a gene in both lists would suggest a functional relationship with DCD.

### 2.3. Third Aim: Analysis of Putative Pathogenetic Overlap between DCD and Ataxia, Chorea, Dystonia and/or Myoclonus

#### Functional Enrichment and Biological Pathway Analysis in the Shared DCD-Associated/MD Gene Co-Expression Network

To explore a putative shared pathogenic background between DCD and the most frequent pediatric movement disorders with a known genetic component, including ataxia, chorea, dystonia and/or myoclonus [27,28], we generated a shared gene co-expression network for these five disorders (hereafter referred as DCD-associated/MD). For this purpose, we used the DCD-associated genes from the literature, as well as ataxia, chorea, dystonia, and myoclonus genes from the Task Force on Genetic Nomenclature in Movement Disorders [47], enriched with 200 predicted genes per each group (Supplementary File S3). Then, we performed shared biological pathway analysis using the meta-analysis function of Metascape. We defined as “overlapping” the biological pathways enriched for the genes associated with DCD and for at least one or more movement disorders (ataxia and/or chorea and/or dystonia and/or myoclonus).

To investigate whether the 200 DCD-predicted genes were linked with movement disorders, we compared these genes with established genes for ataxia, dystonia, myoclonus, chorea, spastic paraplegia, neurodegeneration with brain accumulation, and mixed movement disorders. We selected genes associated with these disorders based on gene lists from our hospital (available at: https://www.umcg.nl/-/afdeling/genetica/aanvragen-genoomdiagnostiek; accessed on 13 March 2023) [48], as well as gene lists from the Task Force on Genetic Nomenclature in Movement Disorders [47].

### 2.4. Statistical Analyses

For all our statistical analyses and graphs, we used GraphPad Prism (version 9.4.0, for Windows; GraphPad Software, San Diego, CA, USA, 2022. Available at: www.graphpad.com). For temporal gene expression analysis, we log10-transformed RPKM RNAseq data and performed both (1) repeated measures one-way ANOVA to compare the expression of the DCD-associated genes among the seven developmental stages within each structure (cerebellum, basal ganglia, and frontal cortex); and (2) repeated measures two-way ANOVA to compare the average gene expression among the seven developmental stages between the cerebellum, basal ganglia, and frontal cortex. For tissue gene expression analysis, we normalized TPM RNAseq data into z-scores and performed a repeated measures one-way ANOVA to compare the average expression of the DCD-associated genes in all available nervous system structures. For both temporal and tissue expression analyses, we corrected for multiple testing using Tukey’s test. Finally, we plotted these data in box-and-whisker plots and bar graphs. We set the significance level to α = 0.05.

## 3. Results

### 3.1. First Aim: Gene Identification in the Literature

#### 12 Genes Associated with DCD in the Literature

We performed a comprehensive literature research in PubMed to identify genes previously associated with DCD. Nine articles out of 268 hits described genetic variants in three genes and 96 loci in patients with a clinical diagnosis of DCD (Supplementary Figure S2, Supplementary Files S4 and S5) [11,22,49,50,51,52,53,54,55]. According to our inclusion criteria (Figure 1), we selected three genes from two case reports of 23 patients with DCD (*ABCC8, KCNJ11, KLF7*) [11,49] and nine genes from seven CNV-loci reported in nine patients with DCD in a CNV-analysis (*CNTN4*, *CTNNA3*, *FHIT*, *GAP43*, *LSAMP*, *PTPRN2 RBFOX1*, *SHANK3*, *VIPR2*) [22]. We reviewed the disease associations of these 12 genes and ascertained that none of them was primarily associated with a known neurological disorder affecting coordination (Supplementary Table S1) [22,49,56,57,58,59,60,61,62,63,64,65,66,67,68]. The nine genes from the seven CNV-loci were associated with DCD-comorbid NDDs. Altogether, these 12 genes thus matched the inclusion criteria and were selected for further in silico analyses (Table 1).

### 3.2. Second Aim: Analysis of Pathogenetic Mechanisms Underlying DCD

#### 3.2.1. Ubiquitous Expression of DCD-Associated Genes in Brain throughout Development

**Figure 2:**
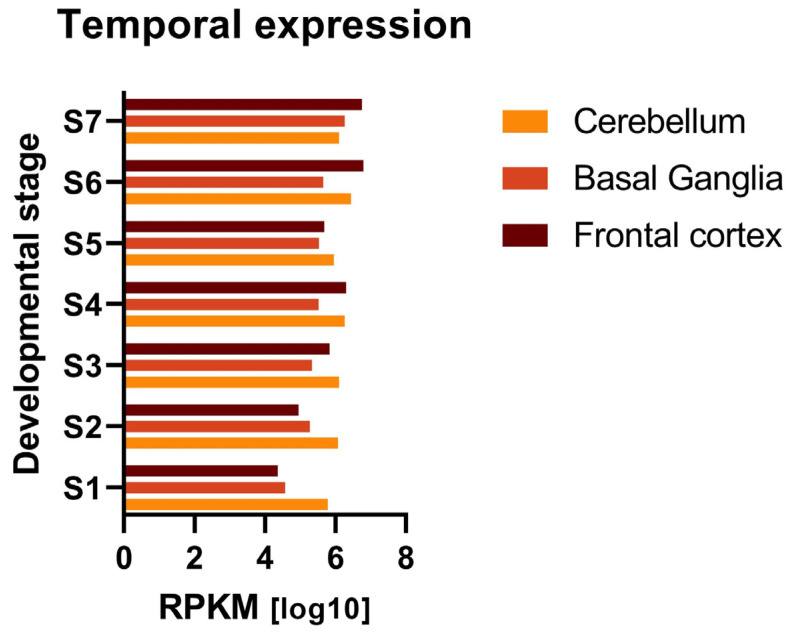
Comparative temporal expression of the 12 DCD-associated genes across different developmental stages in the cerebellum, basal ganglia, and frontal cortex. Log10-transformed mean gene expression is depicted for each developmental stage (S1–S7) in the selected structures. Gene expression was available as reads per kilobase per million (RPKM). Comparison of average gene expression among the developmental stages between the cerebellum, basal ganglia, and frontal cortex showed no statistically significant difference after correcting for multiple comparisons. Mean, range, and standard deviation of each developmental stage in each of the three structures are reported in Supplementary Table S2. RPKM = reads per kilobase per million; S1 = 8–13 postconceptional weeks; S2 = 16–21 postconceptional weeks; S3 = 24–37 postconceptional weeks; S4: 0–1 years; S5 = 2–4 years; S6 = 8–13 years; S7 = 15–19 years.

Using publicly available RNAseq data, we investigated both the temporal- and tissue-specific expression of the 12 DCD-associated genes. First, we analyzed their expression patterns during brain development in the cerebellum, basal ganglia, and frontal cortex across seven developmental stages (S1–S7, Supplementary Figure S1). The average log10 gene expression values of the 12 DCD-associated genes ranged from 4.36 to 6.79, varying per structure and per developmental stage (Supplementary Figure S3, Supplementary Table S2). Comparison of mean gene expression levels across the developmental stages within each structure and between the three anatomical structures showed no statistically significant difference after correcting for multiple comparisons (Supplementary Figure S3, Figure 2).

**Figure 3:**
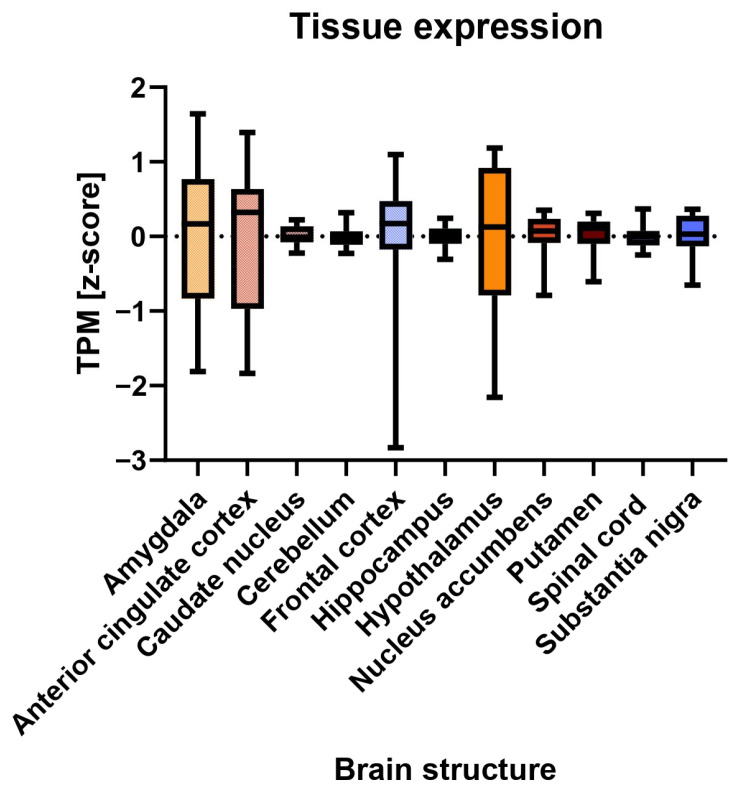
Comparative tissue expression of the 12 DCD-associated genes in central nervous system structures. For each brain structure, the normalized mean gene expression values (z-score) are given as transcript per million (TPM). Comparison of average gene expression among the investigated brain structures showed no statistically significant differences. Mean, range, and standard deviation of the expression data for each brain structure are reported in Supplementary Table S3. TPM = Transcript per Million.

Second, we investigated whether the average expression of the 12 DCD-associated genes was specific for any of the available CNS structures. The mean gene expression levels ranged from −7.50 × 10^−7^ to 8.33 × 10^−8^ in the diverse CNS structures (Supplementary Table S3). There was no significant difference in average gene expression levels among the investigated CNS structures (Figure 3).

#### 3.2.2. Three Main Biological Themes in the DCD-Associated Gene Co-Expression Network

To explore the biological relationship between the 12 DCD-associated genes, we generated a brain-specific DCD-associated gene co-expression network through functional enrichment and then performed biological pathway analysis. Using Metascape, 76 clusters were identified in the DCD-associated gene co-expression network, comprising of 546 biological pathways (Supplementary File S6). Among the top 20 most significantly enriched clusters of biological pathways, we identified three main biological themes based on the parental biological term of each cluster (Table 2). The three themes were: (1) cellular processes, including cell junction organization, regulation of ion transport, cell-cell adhesion, and protein localization to membrane, among others; (2) neural signaling, including modulation of chemical synaptic transmission, synaptic signaling, and regulation of glutamatergic synaptic transmission; and (3) nervous system development, comprising of neuron projection development, L1CAM interactions, and brain development (Table 2, Supplementary File S6). Notably, similar biological pathways and themes were identified using different enrichment analysis programs (Supplementary File S6).

The brain-specific DCD-associated gene co-expression network contained 200 DCD-predicted genes (Supplementary File S2). We also investigated the overlap between the DCD-predicted genes and the genes in the DCD-associated CNV-loci (Supplementary File S5). Only one gene in the CNV-locus 16p11.2, *TLCD3B*, was present in the DCD-associated co-expression network.

### 3.3. Third Aim: Analysis of Putative Pathogenetic Overlap between DCD and Ataxia, Chorea, Dystonia and/or Myoclonus

#### Three Main Biological Themes in the Shared DCD/MD Gene Co-Expression Network

**Figure 4:**
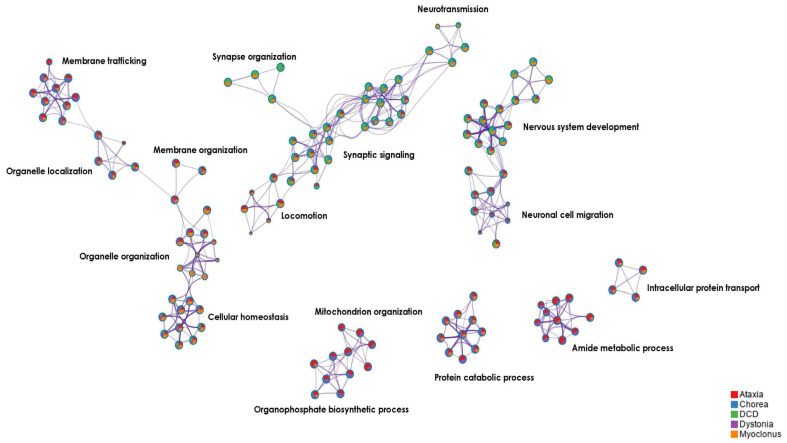
Top significant biological pathways (depicted as clusters) enriched in the shared DCD-associated/MD gene co-expression network. Network plot of the top significant enriched biological pathways, depicted as clusters, in the shared DCD-associated/MD gene co-expression network. Similar biological pathways are grouped in clusters. Clusters are here represented as color-coded pie charts based on their enrichment for each gene group, where red = enriched for the ataxia genes, blue = enriched for the chorea genes, green = enriched for the DCD-associated genes, purple = enriched for the dystonia genes, and orange = enriched for the myoclonus genes. Clusters with similar biological functions are displayed in closer proximity to each other. The name of the cluster with the most significant *p*-value within each group is shown above.

To investigate a putative shared pathogenetic background between DCD and pediatric genetic movement disorders, including ataxia, chorea, dystonia and/or myoclonus, we generated a shared DCD-associated/MD gene co-expression network. Here, we identified 2269 biological pathways that were enriched for genes associated with either DCD, ataxia, chorea, dystonia, myoclonus, or for several disorders simultaneously (Supplementary File S7). A total of 543 biological pathways were enriched for the genes associated with DCD and for at least one or more movement disorders (ataxia and/or chorea and/or dystonia and/or myoclonus). These shared biological pathways were related to (1) neural signaling, such as modulation of chemical synaptic transmission, and trans-synaptic signaling, (2) nervous system development, including neuron projection-, axon-, pallium-, cerebral cortex-, telencephalon development, and (3) cellular processes, such as organelle localization, cellular homeostasis, and cellular transport, among others (Supplementary File S7). A total of 206 of the 543 biological pathways (38%) were uniquely enriched for DCD-associated genes, including pathways related to (1) cellular processes, such as cell-cell interaction, cellular localization, homeostasis, and cellular metabolism, and (2) neurodevelopmental processes, such as dendrite development, postsynaptic density organization, and axonogenesis, among others (Supplementary File S7). The remaining 337 of the 543 biological pathways (62%) overlapped between DCD-associated genes and genes linked to one or more movement disorders (ataxia and/or chorea and/or dystonia and/or myoclonus) (Figure 4).

The largest overlap in biological processes was between DCD-associated genes and myoclonus genes (81 biological pathways) (Supplementary File S7). Moreover, we observed that 12 out of 200 DCD-predicted genes (6%) were established genes for ataxia, dystonia, myoclonus, and spastic paraplegia (Supplementary Table S4).

## 4. Discussion

To the best of our knowledge, this is the first study to comprehensively investigate and compare the pathogenetic mechanisms underlying DCD and those of several movement disorders. Our data show the association of 12 genes with DCD in the literature. These 12 DCD-associated genes are ubiquitously expressed in the central nervous system throughout brain development and are mainly involved in cellular processes, neural signaling, and nervous system development. These results are supportive of a genetic substrate in a subgroup of patients with DCD. Furthermore, the underlying pathogenetic mechanisms of the DCD-associated genes overlap substantially (62%) with those of several movement disorders, including ataxia, chorea, dystonia and/or myoclonus. This implies that the genetic substrate of DCD could be regarded as part of a broader pathogenetic movement disorder spectrum.

### 4.1. The Association of 12 Genes with DCD Suggests the Existence of a Genetic Etiological Subgroup

The first aim of our study was to identify all genes associated with DCD in the literature. By comprehensively reviewing the literature, we included 12 genes whose variants were reported in 32 patients with a clinical diagnosis of DCD according to the DSM-4/5 criteria [11,22,49]. These 12 genes were mainly associated with DCD-comorbid NDDs, such as ADHD and ASD, and with neurological conditions not primarily affecting coordination, such as epilepsy, depression, or schizophrenia. Interestingly, SNPs in *ABCC8* and *KCNJ11* were reported in patients with neonatal diabetes mellitus and DCD [49]. Although no causal correlation was found between their genotypes and neurodevelopmental outcomes, these patients received a clinical diagnosis of DCD according to the DSM-4 criteria [49]. Therefore, following our inclusion criteria, we included these two genes. Some other genes, such as *KCNJ11*, *KLF7* and *VIPR2*, have previously been associated with mild ID (IQ > 55) [22,49,64,65]. Despite ID being an exclusion criterion for DCD, the official guidelines do not specify an IQ cut-off [2,3]. Instead, they indicate that DCD should not be diagnosed when the symptoms can be explained by moderate to severe ID, as defined by the ICD-10 [2,3]. This corresponds to an IQ of <50 [29]. Therefore, because the abovementioned genes were associated with an IQ of >55, we included them in our list. Also, to ascertain that we did not select genes associated with a DCD-like phenotype, we defined the absence of a diagnosis of DCD according to the official DSM-4/5 criteria as an exclusion criterion. In fact, the association of DCD with genes reported with DCD-like phenotypes was unlikely, because these genes were associated with conditions that were exclusion criteria for DCD. This was, for instance, the case for *COL6A1* [24], associated with Bethlem myopathy 1 (OMIM #158810) and Ullrich congenital muscular dystrophy 1 (OMIM #254090), and for *IQSEC1* [25], associated with severe ID (OMIM #618687). As such, we did not include these genes in our list. Altogether, by strictly complying with the official diagnostic criteria for DCD [2,3], we are confident that the 12 included genes are representative of a possible genetic substrate of DCD. These results suggest the existence of a genetic subgroup among the putative etiological causes of DCD.

### 4.2. The Analysis of the Pathogenetic Mechanisms of DCD-Associated Genes Reveals the Lack of Unique Findings

#### 4.2.1. Analogous Expression Patterns of DCD- and Movement Disorder Genes

Our second aim was to expose the pathogenetic mechanisms underlying the 12 DCD-associated genes through the analysis of temporal and tissue gene expression, and of brain-specific biological pathways. The ubiquitous expression of the 12 DCD-associated genes in the CNS during development suggests a role for these 12 genes in the pathogenesis of a central developmental motor disorder, such as DCD. Also, these results indicate the lack of a unique gene expression signature for the 12 DCD-associated genes. In fact, the temporal expression patterns of the 12 DCD-associated genes in the cerebellum, basal ganglia, and frontal cortex were similar to those of ataxia, dystonia, and myoclonus genes in the cortico-basal-ganglia-cerebellar (CBGC) network [35,69,70]. In the literature, the disruption of the CBGC network is described in both DCD [71,72,73,74,75] and in movement disorders, including ataxia, dystonia, and myoclonus [35,69,70,76,77,78,79]. Altogether, the analogous temporal expression patterns of DCD-associated genes and movement disorder genes, and the involvement of the CBGC network in both diagnostic groups, suggest that DCD could be part of a pathogenetic spectrum of movement disorders.

#### 4.2.2. A Putative Novel Role for Contactins and Ankyrins in the Pathogenesis of DCD

In the unique DCD-associated gene co-expression network, we identified three main biological themes, including cellular processes, neural signaling, and nervous system development. These generic biological processes have previously been associated with ataxia, dystonia, and myoclonus [34,35,69,70,78]. Interestingly, two DCD-associated genes, *CNTN4* and *SHANK3*, were present in the most significantly enriched pathways and had overlapping biological functions. As such, they may provide novel insights into the pathogenetic mechanisms of DCD. *CNTN4* and *SHANK3* belong to the contactin subgroup of the immunoglobulin superfamily and the SHANK family, containing multiple ankyrin repeats [80,81,82]. These genes encode neuronal cell adhesion molecules and scaffolding proteins that promote the modulation of neuronal activity, including glutamatergic synaptic excitability, nodal and paranodal organization, and various neurodevelopmental and cellular processes, such as neuron projection development, axono- and synaptogenesis, neurite outgrowth, synaptic growth and maintenance, and protein-protein interaction [56,80,83,84,85]. *SHANK3*-haploinsufficiency is reported in Phelan-McDermid syndrome, a rare condition characterized by ID, hypotonia, global developmental delay, and ASD, among other features [67]. Despite the known involvement of *SHANK3* in this disorder, deletions of the 22q13 locus, associated with this phenotype, encompass a much larger group of genes [68]. Interestingly, both *CNTN4* and *SHANK3* have been associated with ASD [22,56,67,80,85] a very frequent DCD-comorbid NDD (about 47%) [3,20,21]. Moreover, motor coordination deficits and gait abnormalities have been described in mice with biallelic deletions of either *SHANK3* N-terminus or C-terminus [86,87]. Similarly, a quantitative trait locus analysis in BXD recombinant inbred lines of mice with DCD-like phenotypic traits proposed *Cntn6/CNTN6*, a gene adjacent to the region of chromosome 3 where *CNTN4* is located [56], as a candidate gene for the regulation of murine coordination and postural control [87]. Altogether, these findings may indicate a putative novel role for contactins and ankyrins in the pathogenesis of DCD, which might be worth exploring in future studies.

#### 4.2.3. TLCD3B, a New Gene for DCD?

In the DCD-associated gene co-expression network, we also explored the overlap between DCD-predicted genes and genes present in CNV-loci associated with DCD in the literature. Based on our gene co-expression analysis, *TLCD3B* from the DCD-associated CNV-locus 16p11.2 may have a functional relationship with the 12 DCD-associated genes. Notably, we did not include *TLCD3B* in our analyses, because the gene was previously not directly associated with any DCD-comorbid NDD. As such, it did not meet our inclusion criteria. However, both deletions and duplications of the regions of the 16p11.2 locus encompassing *TLCD3B* have previously been associated with various NDDs, such as DCD, ASD, language delay, and different levels of ID [22,52,55], but also with movement disorders causing coordination impairments, including ataxia and dystonia [88]. Given that many genes are found in this locus, and that there was no direct association of *TLCD3B* with movement disorders, we consider *TLCD3B* a putative DCD-associated gene. *TLCD3B* encodes the TLC domain-containing protein 3B and functions as a ceramide synthetase, mediating stress responses and aiding survival of retinal cells [89]. Bulk expression of *TLCD3B* in the CNS is the highest in the cerebellum [37], indicating a possible crucial role for this gene in this brain structure. Recently, homozygous variants in *TLCD3B* have been associated with cone-rod dystrophy 22, a retinal condition leading to progressive central vision loss [89]. Possibly, heterozygous variants in *TLCD3B* could induce a different phenotype than vision loss, such as coordination impairments. This should be investigated in future studies.

### 4.3. The Analysis of a Putative Shared Pathogenetic Background Reveals a Substantial Overlap between DCD and Several Movement Disorders

#### 4.3.1. A Common Pathogenetic Substrate between DCD and Movement Disorders

Our third aim was to explore a putative shared pathogenetic background between DCD and movement disorders, by analyzing a shared DCD-associated/MD gene co-expression network. We identified a 62% overlap in biological pathways between DCD-associated genes and genes linked to at least one or several movement disorders, such as ataxia, chorea, dystonia and/or myoclonus. These overlapping biological pathways were related to the same three biological themes of the unique DCD-associated gene co-expression network, including neural signaling, nervous system development, and cellular processes. Interestingly, the remaining 38% of biological pathways enriched only for DCD-associated genes were related to similar biological themes, including cellular- and neurodevelopmental processes. Therefore, these findings indicate the absence of distinctive biological themes for the DCD-associated genes in relation to the investigated movement disorders. This suggests that the biological overlap between DCD and the abovementioned movement disorders could exceed the reported 62%. An overlapping pathogenetic substrate is also suggested by the fact that 6% (12/200) of the functionally DCD-predicted genes are established movement disorder genes, mainly linked with ataxia and myoclonus. Until now, neither the large biological overlap we observed between myoclonus- and DCD-associated genes, nor a putative phenotypic association between these two disorders have been reported in literature.

#### 4.3.2. Diagnostic Implications of a Shared Pathogenetic Spectrum and Future Perspectives

Although the presence of movement disorders is an exclusion criterion for DCD [2,3], mild clinical features of ataxia, dystonia, and/or chorea have previously been described in patients with a diagnosis of DCD according to the official diagnostic criteria [6,11]. In these patients, the clinical distinction between DCD and mild movement disorders might lead to diagnostic delay. Considering our overlapping pathogenetic findings between the 12 DCD-associated genes and the investigated movement disorders, the question arises whether DCD exists as a unique diagnostic entity or as the milder end of a broader movement disorder spectrum. Based on our findings, we suggest considering the genetic subgroup of DCD as part of a pathogenetic movement disorder spectrum. This would have important diagnostic implications, such as the inclusion of mild movement disorder features as part of the motor phenotype in DCD. In future studies, we aim to further investigate the implications of this paper by thoroughly phenotyping a cohort of putative DCD patients. This may hopefully unravel the diagnostic conundrum existing between DCD and movement disorders. Additionally, in this cohort, genetic diagnostic analysis of the 12 DCD-associated genes might provide further evidence for an underlying genetic substrate of DCD.

### 4.4. Limitations of This Study

We recognize several limitations to this study. First, the list of genes reported in the literature in association with DCD was short, therefore our set of genes may be incomplete. So far, two genome-wide association studies have been performed in patients with DCD-like phenotypes, but none in patients with a diagnosis of DCD. This is likely because diagnostic genetic testing is still not routinely performed in patients with an official diagnosis of DCD. We therefore hypothesize that new genetic associations or gene mutations will likely be exposed in the future. However, by comprehensively reviewing the literature and strictly complying with the diagnostic criteria for DCD, we are confident that our findings are representative of the current knowledge. Second, we used in silico analyses to expose the underlying pathogenetic mechanisms. We are aware that although in silico analyses may be helpful to detect patterns of similarities within a large set of genes, these strategies might overlook specific gene characteristics, such as particular biological functions or molecular mechanisms.

## 5. Conclusions

In the present study, we aimed to explore the pathogenetic mechanisms underlying DCD-associated genes in relation to those of movement disorders with a known genetic component. Our findings indicate the existence of a genetic etiological subgroup in DCD, whose pathogenetic mechanisms overlap substantially with those of movement disorders. These data suggest that the genetic subgroup of DCD belongs to a broader pathogenetic movement disorder spectrum. Our study implies that there is the need for thorough phenotyping of DCD patients, including the evaluation of mild movement disorder features.

## Figures and Tables

**Table 1:** Final list of 12 genes reported in the literature in 32 patients with DCD.

Gene	Genetic Variant Information	Clinical Information
*ABCC8*	This information can be found in the manuscript of Busiah et al. (2013), see Supplementary Tables 3 and 7 [49]	11 patients with nDM and DCD
*CNTN4*	CNV (deletion) in locus 3p26.3, unknown inheritance [22]	1 patient with isolated DCD
*CTNNA3*	CNV (deletion) in locus 10q21.3, paternally inherited [22]	1 patient with isolated DCD *^1^ 1 patient with DCD and ADHD
*FHIT*	CNVs (deletion) in locus 3p14.2, unknown inheritance [22]	2 patients with isolated DCD
*GAP43*	CNV (deletion) in locus 3q13.31, de novo [22]	1 patient with DCD and ADHD *^2^
*KCNJ11*	This information can be found in in the manuscript of Busiah et al. (2013), see Supplementary Tables 2 and 8 [49]	11 patients with nDM and DCD
*KLF7*	Not available [11]	1 patient with DCD
*LSAMP*	CNV (deletion) in locus 3q13.31, de novo [22]	1 patient with DCD and ADHD *^2^
*PTPRN2*	CNV (duplication) in locus 7q36.3, maternally inherited [22]	1 patient with DCD, ADHD, and RD
*RBFOX1*	CNV (deletion) in locus 16p13.3, maternally inherited [22]	1 patient with isolated DCD *^1^
*SHANK3*	CNV (duplication) in locus 22q13.33, maternally inherited [22]	1 patient with isolated DCD
*VIPR2*	CNV (deletion) in locus 7q36.3, maternally inherited [22]	1 patient with DCD and ADHD

12 genes reported in the literature in 32 patients with a clinical diagnosis of DCD. These 12 genes matched all the inclusion criteria (Figure 1) and were therefore included in our in silico analyses. The genetic information of SNP variants in the *ABCC8* and *KCNJ11* genes can be found in the Supplementary Tables 2, 3, 7 and 8 of the manuscript of Busiah et al. (2013) [49]. Further genetic information regarding the CNVs (chromosome, affected region of the locus, size of the CNV, and genes found in that region) can be found in Supplementary File S5 of this manuscript. The numbers in square brackets correspond to the references cited in the manuscript. nDM: neonatal diabetes mellitus; DCD: developmental coordination disorder; ADHD: attention deficit/hyperactivity disorder; RD: reading disorder. *^n^ Same patient as indicated by the same number.

**Table 2:** Top 20 significant biological clusters enriched in the DCD-associated gene co-expression network.

Biological Cluster	Parental Biological Term	*p*-Value (Log10)
Modulation of chemical synaptic transmission (GO:0050804)	Synaptic signaling	−26.82
Synaptic signaling (GO:0099536)	Synaptic signaling	−21.91
Cell junction organization (GO:0034330)	Cellular process—Cellular component organization	−15.87
Neuronal system (R-HSA-112316)	Synaptic signaling—Chemical synaptic transmission	−14.69
Behavior (GO:0007610)	Multicellular organismal process	−13.99
Regulation of cell projection organization (GO:0031344)	Cellular process—Cellular component organization	−13.61
Neuron projection development (GO:0031175)	Nervous system development	−12.77
Regulation of ion transport (GO:0043269)	Cellular process—Transport	−9.48
Protein localization to synapse (GO:0035418)	Cell process—Cellular localization	−8.94
L1CAM interactions (R-HSA-373760)	Nervous system development—Axon guidance	−8.89
Metal ion transport (GO:0030001)	Cellular process—Transport	−7.57
Actin filament-based process (GO:0030029)	Cellular process	−7.00
Cell-cell adhesion (GO:0098609)	Cellular process—Cell adhesion	−6.35
Action potential (GO:0001508)	Biological regulation—Regulation of biological quality	−6.30
Neuromuscular process (GO:0050905)	Nervous system process	−5.94
Brain development (GO:0007420)	Nervous system development	−5.42
Protein localization to membrane (GO:0072657)	Cellular process—Cellular localization	−5.32
Calcium-ion regulated exocytosis (GO:0017156)	Cellular process—Export from cell	−5.32
Regulation of glutamatergic synaptic transmission (GO:0051966)	Synaptic signaling	−4.91
Locomotory behavior (GO:0007626)	Multicellular organismal process—Behavior	−4.78

Top 20 most significantly enriched clusters of biological pathways for the 12 DCD-associated genes. In Metascape, biological pathways with similar biological functions are clustered together. The biological pathway with the highest *p*-value becomes the cluster representative, after which the cluster is named. A unique biological pathway identifier is given in brackets for each pathway (i.e., GO:.. and R-HSA-..); more information can be found online: https://geneontology.org/docs/GO-term-elements and https://reactome.org/ (both accessed on 30 May 2022). The *p*-value for each cluster is expressed in log10, corrected for multiple testing. Parental biological terms are given for each cluster, indicating the broader terms of which the specific biological pathway (i.e., cluster representative) is part, i.e., “cell junction organization” is a form of “cellular component organization”, which in turn is a “cellular process”. This information can be found online: https://geneontology.org/docs/GO-term-elements and https://www.informatics.jax.org/vocab/gene_ontology (both accessed on 30 May 2022). Based on the parental biological terms, we identified three main biological themes: (1) cellular processes, (2) neural signaling, and (3) nervous system development. The complete list of enriched clusters and biological pathways is shown in Supplementary File S6.

## Data Availability

Publicly available datasets were analyzed in this study. These data can be found here: (1) BrainSpan: https://www.brainspan.org/static/home; (2) Genotype-Tissue Expression (GTEx) Portal: https://www.gtexportal.org; (3) gProfiler: https://biit.cs.ut.ee/gprofiler/gost; (4) Gene panels from the University Medical Center Groningen: https://www.umcg.nl/-/afdeling/genetica/aanvragen-genoomdiagnostiek; (5) Metabrain: https://network.metabrain.nl; (6) Metascape: http://metascape.org; (7) ToppGene Suite: https://toppgene.cchmc.org/enrichment.jsp; (8) UCSC Genome Browser: http://genome.ucsc.edu.

## Supplementary Materials

The following supporting information can be downloaded at: https://www.mdpi.com/article/10.3390/brainsci13121625/s1, Figure S1: Reclassification of seven developmental stages; Figure S2: Overview of the results of the literature research; Figure S3: Temporal gene expression of DCD-associated genes in different developmental stages of the cerebellum, basal ganglia, and frontal cortex; Table S1: Gene association to disease; Table S2: Descriptive statistics of temporal gene expression in different developmental stages of the cerebellum, basal ganglia, and frontal cortex; Table S3: Descriptive statistics of tissue gene expression in different structures of the central nervous system; Table S4: Association of DCD-predicted genes with known movement disorders; File S1: Search string for PubMed; File S2: 200 genes predicted to be functionally similar to the DCD-associated genes (DCD-predicted genes); File S3: Shared DCD-associated/MD gene co-expression network; File S4: Literature research results; File S5: CNV-loci associated with DCD in the literature; File S6: Biological pathway analysis results for the DCD-associated gene co-expression network; File S7: Biological pathway analysis results for the DCD-associated/MD gene co-expression network.

## Abbreviations

DCD	Developmental Coordination Disorder
ADHD	Attention Deficit Hyperactivity Disorder
ASD	Autism-Spectrum Disorder
ID	Intellectual disability
NDDs	Neurodevelopmental disorders
CNV	Copy-number variation
SNP	Single nucleotide polymorphism
OMIM	Online Mendelian Inheritance in Man
RPKM	Reads per kilobase per million
RNAseq	RNA sequencing
CNS	Central nervous system
TPM	Transcript per million
MD	Movement disorder(s)
nDM	Neonatal diabetes mellitus
RD	Reading disorder
CBGC	Cortico-basal ganglia-cerebellar (network)
