# Supplementary material for: Pathogenetic Insights into Developmental Coordination Disorder Reveal Substantial Overlap with Movement Disorders
Source: Brain Sci. 2023 Nov 23;13(12):1625. doi: 10.3390/brainsci13121625 (PMC10741651; doi:10.3390/brainsci13121625)
Supplement: Supplementary file 1 [file brainsci-13-01625-s001.zip › Supplementary Figure S1. Developmental stages.pptx]

## Slide 1
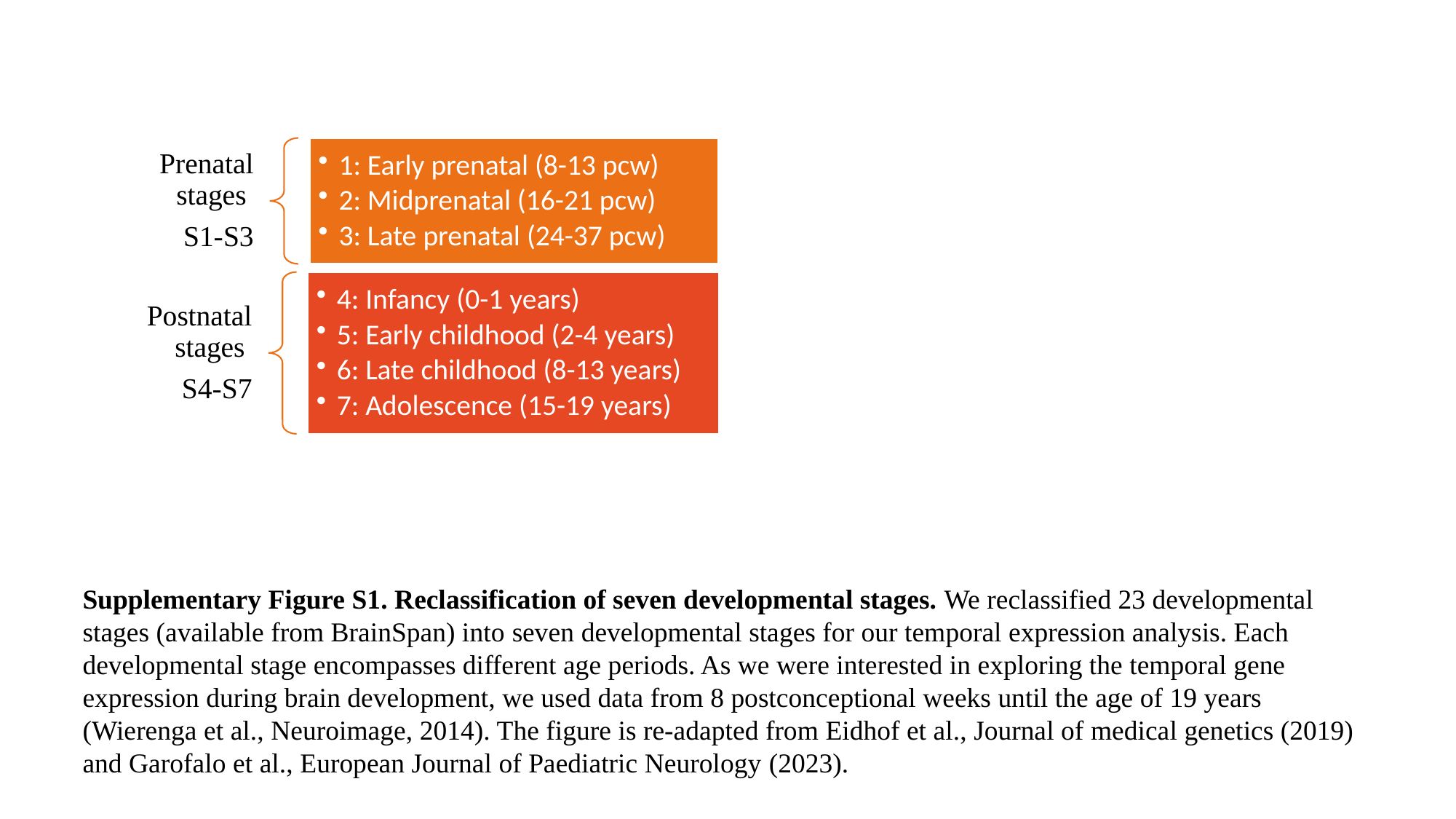

Supplementary Figure S1. Reclassification of seven developmental stages. We reclassified 23 developmental stages (available from BrainSpan) into seven developmental stages for our temporal expression analysis. Each developmental stage encompasses different age periods. As we were interested in exploring the temporal gene expression during brain development, we used data from 8 postconceptional weeks until the age of 19 years (Wierenga et al., Neuroimage, 2014). The figure is re-adapted from Eidhof et al., Journal of medical genetics (2019) and Garofalo et al., European Journal of Paediatric Neurology (2023).
