# Supplementary material for: Pathogenetic Insights into Developmental Coordination Disorder Reveal Substantial Overlap with Movement Disorders
Source: Brain Sci. 2023 Nov 23;13(12):1625. doi: 10.3390/brainsci13121625 (PMC10741651; doi:10.3390/brainsci13121625)
Supplement: Supplementary file 1 [file brainsci-13-01625-s001.zip › Supplementary Figure S2. Overview of results of literature search.pptx]

## Slide 1
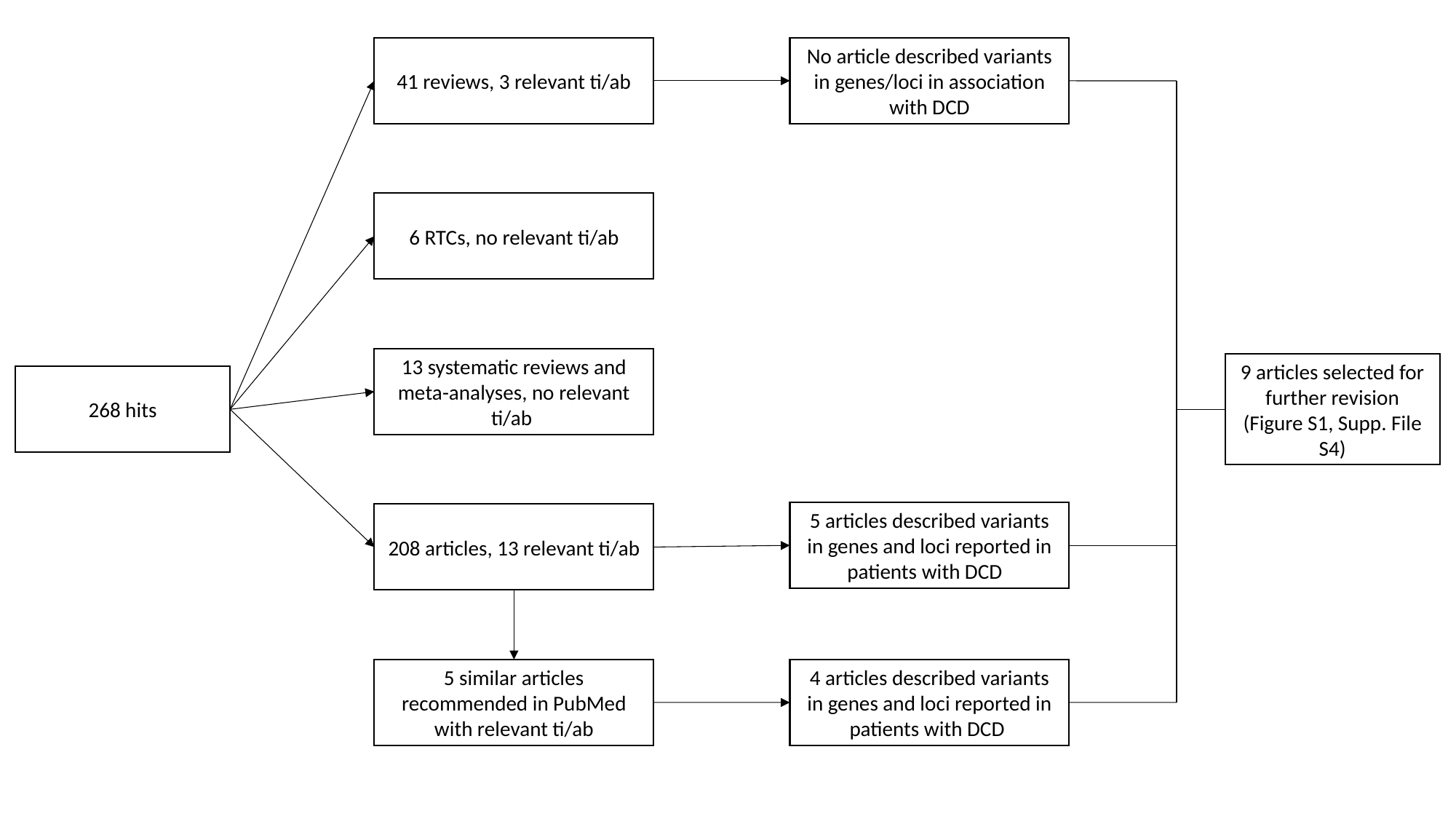

41 reviews, 3 relevant ti/ab
No article described variants in genes/loci in association with DCD
6 RTCs, no relevant ti/ab
13 systematic reviews and meta-analyses, no relevant ti/ab
9 articles selected for further revision (Figure S1, Supp. File S4)
268 hits
5 articles described variants in genes and loci reported in patients with DCD
208 articles, 13 relevant ti/ab
5 similar articles recommended in PubMed with relevant ti/ab
4 articles described variants in genes and loci reported in patients with DCD
