# Supplementary material for: Pathogenetic Insights into Developmental Coordination Disorder Reveal Substantial Overlap with Movement Disorders
Source: Brain Sci. 2023 Nov 23;13(12):1625. doi: 10.3390/brainsci13121625 (PMC10741651; doi:10.3390/brainsci13121625)
Supplement: Supplementary file 1 [file brainsci-13-01625-s001.zip › Supplementary Figure S3. Temporal gene expression in cerebellum, basal ganglia and frontal cortex.pptx]

## Slide 1
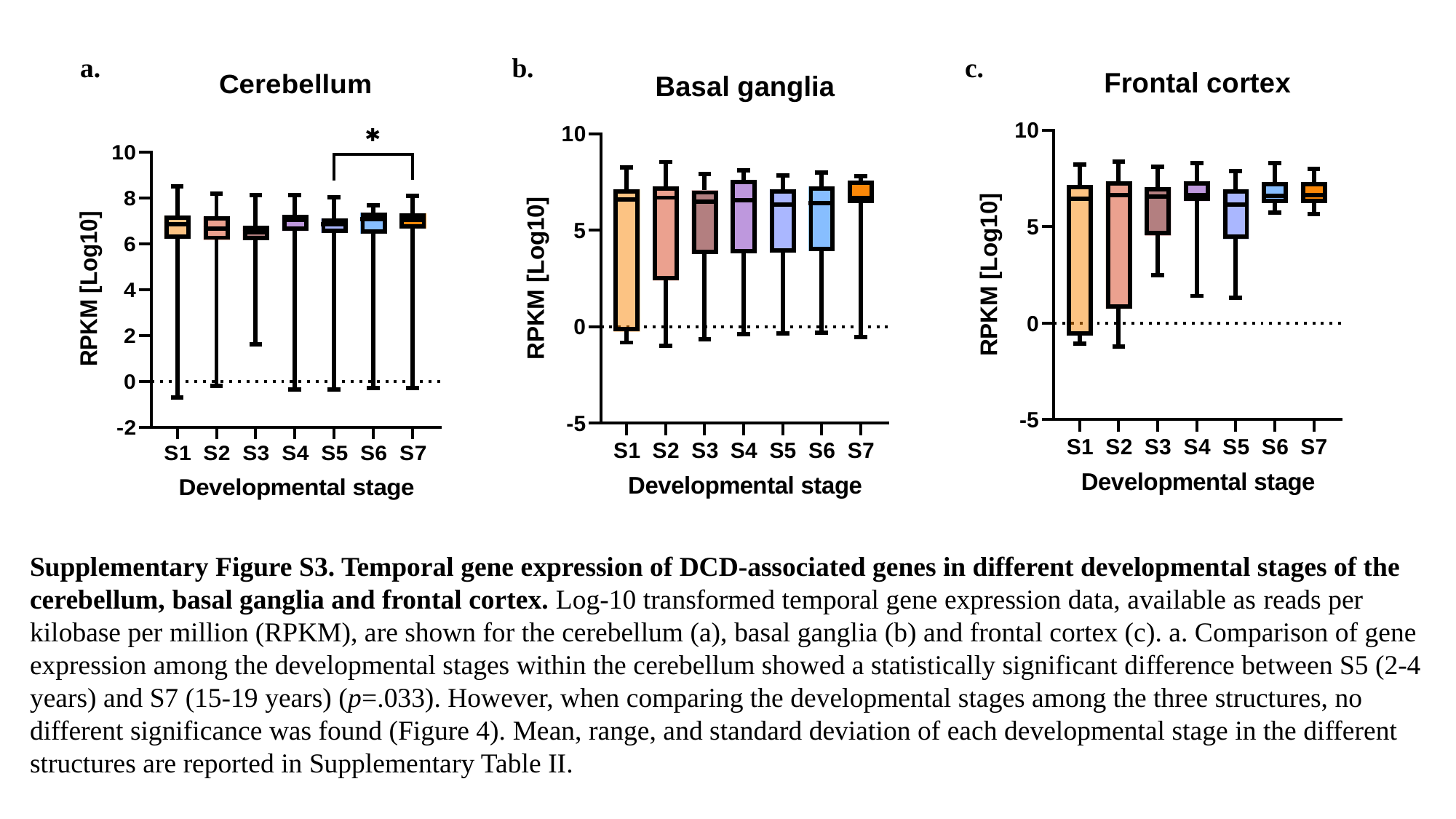

b.
c.
a.
Supplementary Figure S3. Temporal gene expression of DCD-associated genes in different developmental stages of the cerebellum, basal ganglia and frontal cortex. Log-10 transformed temporal gene expression data, available as reads per kilobase per million (RPKM), are shown for the cerebellum (a), basal ganglia (b) and frontal cortex (c). a. Comparison of gene expression among the developmental stages within the cerebellum showed a statistically significant difference between S5 (2-4 years) and S7 (15-19 years) (p=.033). However, when comparing the developmental stages among the three structures, no different significance was found (Figure 4). Mean, range, and standard deviation of each developmental stage in the different structures are reported in Supplementary Table II.
