# Supplementary material for: Pathogenetic Insights into Developmental Coordination Disorder Reveal Substantial Overlap with Movement Disorders
Source: Brain Sci. 2023 Nov 23;13(12):1625. doi: 10.3390/brainsci13121625 (PMC10741651; doi:10.3390/brainsci13121625)
Supplement: Supplementary file 1 [file brainsci-13-01625-s001.zip › Supplementary File S1. Search strategy in PubMed.pdf]

Supplementary File S1. Search string for PubMed (accessed in May 2022).

("Motor Skills Disorders"[Mesh] OR developmental coordination disorder*[tiab] developmental coordination disorder*[Text Word] OR DCD[tiab] OR motor skills disorder*[tiab] OR "Motor skills disorders/ physiopathology"[Mesh] OR "Motor Skills Disorders/genetics"[Mesh]) AND ("Neurodevelopmental Disorders"[Mesh] OR "Neurodevelopmental Disorders/genetics"[Mesh]) AND ("Genome, Human" [Mesh] OR "Genome, Human/genetics"[Mesh] OR "Genetic loci" [Mesh] OR "Genomic Structural Variation"[Mesh] OR "Genetic Predisposition to Disease"[Mesh] OR "Genetic Association Studies"[Mesh] OR "Genome-Wide Association Study"[Mesh] OR gene*[tiab] OR locus[tiab] OR loci[tiab] OR copy number variation*[tiab] OR CNV* [tiab] OR "DNA Copy Number Variations*"[Mesh] OR Copy-number variant [tiab] OR "Chromosome Deletion"[Mesh] OR "Chromosome Duplication" [Mesh] OR "Chromosome Disorders/genetics"[Mesh]) AND ("Child"[Mesh] OR "Adolescent"[Mesh] OR child*[tiab] OR pediatr*[tiab] OR paediatr*[tiab] OR adolescen*[tiab] OR youth[tiab] OR teenager*[tiab] OR infan*[tiab] OR "Humans"[Mesh]) NOT (("Animals"[Mesh] NOT "Humans"[Mesh]) OR animal*[ti] OR mouse[ti] OR mice[ti])
