# Supplementary material for: Pathogenetic Insights into Developmental Coordination Disorder Reveal Substantial Overlap with Movement Disorders
Source: Brain Sci. 2023 Nov 23;13(12):1625. doi: 10.3390/brainsci13121625 (PMC10741651; doi:10.3390/brainsci13121625)
Supplement: Supplementary file 1 [file brainsci-13-01625-s001.zip › Supplementary File S2. 200 DCD-predicted genes .pdf]

Supplementary File S2. 200 genes predicted to be functionally similar to the DCD-associated genes (DCD-predicted genes). These genes were procedurally retrieved for the enrichment of the DCD gene co-expression network using the network function of the program MetaBrain (https://network.metabrain.nl).

TSPAN7	RUNDC3A	CELSR3
NRXN1	SYT3	GRAMD1B
SNAP91	CRMP1	CNTN1
CELF3	MAPK8IP2	CAMTA2
UNC13A	KIF1A	TRIM67
JPH4	SPTB	PSD
EEF1A2	MGAT5B	LRRTM1
KCNQ2	RIMS3	ATP1A3
BRSK2	PPFIA3	DUSP8
SEMA6B	NRXN2	BCR
ATP2B2	RIT2	NEURL1
JPH3	BCAS1	NTRK2
PHACTR3	ASTN1	CHRNA4
CTNNA2	TMEM151B	PAIP2B
TLCD3B	RTN2	KCNK12
IGSF21	NMNAT2	DLG4
CADM2	FBXL16	AGAP2
NTM	CDH22	MAPT-AS1
HECW1	RNF165	FGF12
SEPTIN3	CELF4	IQSEC2
AC005696.4	CSMD1	MAPT
CPLX1	ATCAY	TNR
CACNA1A	ELAVL3	PTPRN
ADD2	AMER3	CYFIP2
SCG3	PPP2R2C	INA

GRIK2	DENND6B	KCNQ3
IQSEC3	SPTSSB	KNDC1
SCRT1	SPATA20	RAB3C
PPP1R1A	CHGB	RAB15
CIT	RIMS2	MAP3K10
KCND1	CACNA1I	NSG1
SYBU	VAT1L	MAP1A
SVOP	BSN	RASSF2
CARMIL2	GSK3B	PAK6
SEZ6L	GDAP1L1	SCN3B
RASGEF1C	LINGO1	LINC00689
PIK3R1	MCF2L	ANK2
FHDC1	PTK2B	GGTA1P
SLITRK3	ZNF385B	CAMKK2
ADAM22	SLC8A2	APC2
MGLL	SEPTIN5	SLC8A3
DEAF1	TMEM63C	FRRS1L
DBNDD1	CAMK1D	DMTN
CPLX2	NR2F1-AS1	SCN2A
PLCB1	ACTL6B	TMOD2
TNPO2	PAK5	PPFIA4
PLCXD2	FBXO41	CADM3
CAMSAP3	PITPNC1	AL356056.2
KCNT1	CSMD3	PIP5K1C
L1CAM	SH3GL2	TMEM35A
SPTBN1	HCN2	DPYSL3
KIF3C	CKMT1B	DLGAP4
DLGAP3	KCND2	ADAM23

MAST1	CPE	EXTL1
MAP3K13	ARHGAP39	PITPNM1
MAP1B	APBA1	CA10
NRSN2	STX1B	CHRNB2
FAIM2	AOAH	STXBP1
MYT1L	CELF5	LUZP2
JAZF1	FAM189A1	PITPNM3
DNER	KLF6	HRH3
RNF150	SBK1	RIMS4
CNTNAP5	ENPP5	SNPH
CADPS	SMPD3	SLC6A17
TMCC3	TMEM271	ARHGDIG
DCX	PALM	SNX29
PDZD4	SGSM1	
