# Supplementary material for: Pathogenetic Insights into Developmental Coordination Disorder Reveal Substantial Overlap with Movement Disorders
Source: Brain Sci. 2023 Nov 23;13(12):1625. doi: 10.3390/brainsci13121625 (PMC10741651; doi:10.3390/brainsci13121625)
Supplement: Supplementary file 1 [file brainsci-13-01625-s001.zip › Supplementary Table S1. Association to disease of DCD genes.pdf]

Supplementary Table S1. Gene association to disease.

Gene	Disease associations
ABCC8	Developmental delay, epilepsy, and neonatal diabetes (DEND) syndrome [49]
CNTN4	ASD [22,56]<br>Candidate gene for SCA16 in a Japanese family (most likely only ascertained to this family, not confirmed in the Japanese population) [57,58]<br>One patient with bilateral optical nerve aplasia [59]<br>Three patients with speech impairment, motor, and cognitive delay; two of these patients had seizures [60]
CTNNA3	ASD and Tourette syndrome [22]<br>Late-onset Alzheimer's disease in women [61]<br>Three patients from one family with essential tremor (rare in the general population) [62]
FHIT	ASD and Tourette syndrome [22]<br>Major depressive disorder [63]
GAP43	Schizophrenia, hypotonia, motor and language delays and different behavioral issues [22]
KCNJ11	Moderate intellectual disability (IQ 40-55), DEND, impaired visuomotor integration, ASD, and ADHD in V59M mutations. Milder features in non-V59M mutations. The majority of the patients described with a mutation in KCNJ11 and a DCD diagnosis had non-V59M mutations [49,64]
KLF7	Candidate gene for 2q33.3q34 deletion (intellectual disability is mainly mild in these patients, with IQ scores >55) [65]
LSAMP	Schizophrenia, hypotonia, motor and language delays, and different behavioral issues [22]<br>Candidate gene for Meniere's disease in one family [66]
PTPRN2	ADHD [22]
RBFOX1	ASD, ADHD and epilepsy [22]
SHANK3	ASD [22]<br>Different neurodevelopmental disorders, including ADHD, intellectual disability, learning impairments, oppositional behavioral disorder, epilepsy [67]<br>Haploinsufficiency causes Phelan-McDermid syndrome [68]
VIPR2	Schizophrenia, ADHD, other cognitive and behavioral phenotypes [22]

List of 12 DCD-associated genes and their disease associations in the literature. References can be found below and in the main text. Numbers in square brackets indicate the corresponding reference in the main text. DEND: developmental delay, epilepsy, and neonatal diabetes; SCA16: spinocerebellar ataxia type 16; ASD: autism-spectrum disorder; IQ: intelligence quotient; ADHD: attention deficit/hyperactivity disorder; DCD: developmental coordination disorder.

References

22. Mosca, S.J.; Langevin, L.M.; Dewey, D.; Innes, A.M.; Lionel, A.C.; Marshall, C.C.; Scherer, S.W.; Parboosingh, J.S.; Bernier, F.P. Copy-number variations are enriched for neurodevelopmental genes in children with developmental coordination disorder. *J. Med. Genet.* **2016**, *53*, 812–819.
49. Busiah, K.; Drunat, S.; Vaivre-Douret, L.; Bonnefond, A.; Simon, A.; Flechtner, I.; Gérard, B.; Pouvreau, N.; Elie, C.; Nimri, R.; et al. Neuropsychological dysfunction and developmental defects associated with genetic changes in infants with neonatal diabetes mellitus: A prospective cohort study. *Lancet Diabetes Endocrinol.* **2013**, *1*, 199–207.
56. Zuko, A.; Kleijer, K.T.E.; Oguro-Ando, A.; Kas, M.J.H.; van Daalen, E.; van der Zwaag, B.; Burbach, J.P.H. Contactins in the neurobiology of autism. *Eur. J. Pharmacol.* **2013**, *719*, 63–74
57. Miura, S.; Shibata, H.; Furuya, H.; Ohyagi, Y.; Osoegawa, M.; Miyoshi, Y.; Matsunaga, H.; Shibata, A.; Matsumoto, N.; Iwaki, A.; et al. The contactin 4 gene locus at 3p26 is a candidate gene of SCA16. *Neurology* **2006**, *67*, 1236-1241.

58. Tanaka, E.: Maruyama, H.; Morino, H.; Nakajima, E.: Kawakami, H. The CNTN4 c.4256C> T mutation is rare in Japanese with inherited spinocerebellar ataxia. *J. Neurol. Sci.* **2008**, *266*, 180-181.
59. Prasov, L.; Masud, T.; Khaliq, S.; Mehdi, S.Q.; Abid, A.; Oliver, E.R.; Silva, E.D.; Lewanda, A.; Brodsky, M.C.; Borchert, M.; et al. ATOH7 mutations cause autosomal recessive persistent hyperplasia of the primary vitreous. *Hum. Mol. Genet*. **2012**, *21*, 3681–3694.
60. Zhang, S.Q.; Fleischer, J.; Al-Kateb, H.; Mito, Y.; Amarillo, I.; Shinawi, M. Intragenic CNTN4 copy number variants associated with a spectrum of neurobehavioral phenotypes. *Eur. J. Med. Genet.* **2020**, *63*, 103736.
61. Miyashita, A.; Arai, H.; Asada, T.; Imagawa, M.; Matsubara, E.; Shoji, M.; Higuchi, S.; Urakami, K.; Kakita, A.; Takahashi, H.; et al. Genetic association of CTNNA3 with late-onset Alzheimer's disease in females. *Hum. Mol. Genet*. **2007**, *16*, 2854-69.
62. Houle, G.; Ambalavanan, A.; Schmouth, J.F.; Leblond, C.S.; Spiegelman, D.; Laurent, S. B.; Bourassa, C.V.; Grayson, C.; Panisset, M.; Chouinard, S.; et al. No rare deleterious variants from STK32B, PPARGC1A, and CTNNA3 are associated with essential tremor. *Neurol. Genet*. **2017**, *3*, e195.
63. Direk, N.; Williams, S.; Smith, J.A.; Ripke, S.; Air, T.; Amare, A.T.; Amin, N.; Baune, B.T.; Bennett, D.A.; Blackwood, D.H.R.; et al. An Analysis of Two Genome-wide Association Meta-analyses Identifies a New Locus for Broad Depression Phenotype. *Biol. Psychiatry*. **2017**, *82*, 322–329.
64. Slingerland, A.S.; Nuboer, R.; Hadders-Algra, M.; Hattersley, A.T.; Bruining, G.J. Improved motor development and good long-term glycaemic control with sulfonylurea treatment in a patient with the syndrome of intermediate developmental delay, early-onset generalised epilepsy and neonatal diabetes associated with the V59M mutation in the KCNJ11 gene. *Diabetologia* **2006**, *49*, 2559–2563.
65. Powis, Z.; Petrik, I.; Cohen, J.S.; Escolar, D.; Burton, J.; van Ravenswaaij-Arts, C.M.A.; Sival, D.A.; Stegmann, A.P.A.; Kleefstra, T.; Pfundt, R.; et al. De novo variants in KLF7 are a potential novel cause of developmental delay/intellectual disability, neuromuscular and psychiatric symptoms. *Clin. Genet.* **2018**, *93*, 1030-1038.
66. Mehrjoo, Z.; Kahrizi, K.; Mohseni, M.; Akbari, M.; Arzhangi, S.; Jalalvand, K.; Najmabadi, H.; Farhadi, M.; Mohseni, M.; Asghari, A.; et al. Limbic System Associated Membrane Protein Mutation in an Iranian Family Diagnosed with Ménière’s Disease. *Arch. Iran Med*. **2020**, *23*, 319-325.
67. Woike, D.; Wang, E.; Tibbe, D.; Hassani Nia, F.; Failla, A.V.; Kibæk, M.; Overgård, T.M.; Larsen, M.J.; Fagerberg, C.R.; Barsukov, I.; et al. Mutations affecting the N-terminal domains of SHANK3 point to different pathomechanisms in neurodevelopmental disorders. *Sci. Rep*. **2022**, *12*, 902.
68. Phelan, K.; McDermid, H.E. The 22q13.3 Deletion Syndrome (Phelan-McDermid Syndrome). *Mol. Syndromol.* **2012**, *2*, 186–201.
