# Supplementary material for: Pathogenetic Insights into Developmental Coordination Disorder Reveal Substantial Overlap with Movement Disorders
Source: Brain Sci. 2023 Nov 23;13(12):1625. doi: 10.3390/brainsci13121625 (PMC10741651; doi:10.3390/brainsci13121625)
Supplement: Supplementary file 1 [file brainsci-13-01625-s001.zip › Supplementary Table S2. Descriptives of temporal gene expression data.pdf]

Supplementary Table S2. Descriptive statistics of temporal gene expression in different developmental stages of the cerebellum, basal ganglia and frontal cortex.

**Developmental stage**	**Cerebellum** Mean [range]; SD	**Basal ganglia** Mean [range]; SD	**Frontal Cortex** Mean [range]; SD
S1	5.78 [-0.69, 8.50]; 3.03	4.57 [-0.82, 8.27]; 3.69	4.36 [-1.04, 8.21]; 3.87
S2	6.07 [-0.2, 8.20]; 2.21	5.27 [-0.99, 8.54]; 3.30	4.95 [-1.21, 8.38]; 3.65
S3	6.10 [1.60, 8.14]; 1.67	5.33 [-0.66, 7.93]; 2.98	5.83 [2.51, 8.11]; 1.82
S4	6.27 [-0.34, 8.15]; 2.31	5.53 [-0.38, 8.09]; 3.03	6.31 [1.42, 8.32]; 1.87
S5	5.96 [-0.36, 8.04]; 2.56	5.53 [-0.34, 7.83]; 2.40	5.7 [1.33, 7.91]; 1.82
S6	6.45 [0.3, 7.69]; 2.17	5.66 [-0.30, 8]; 2.45	6.79 [5.73, 8.32]; 0.74
S7	6.10 [-0.29, 8.09]; 2.53	6.26 [-0.55, 7.79]; 2.27	6.75 [5.66, 8.01]; 0.70

Footnote. Mean, range (minimum, maximum), and standard deviation (SD) of normalized (log10-transformed) gene expression data are given per each developmental stage in the cerebellum, basal ganglia, and frontal cortex. Values were calculated based on the means of individual expression data of the 12 DCD-associated genes. Gene expression data were available as reads per kilobase per million. Developmental stages: S1: 8-13 postconceptional weeks (pcw); S2: 16-21 pcw; S3: 24-37 pcw; S4: 0-1 year; S5: 2-4 years; S6: 8-13 years; S7: 15-19 years.
