# Supplementary material for: Pathogenetic Insights into Developmental Coordination Disorder Reveal Substantial Overlap with Movement Disorders
Source: Brain Sci. 2023 Nov 23;13(12):1625. doi: 10.3390/brainsci13121625 (PMC10741651; doi:10.3390/brainsci13121625)
Supplement: Supplementary file 1 [file brainsci-13-01625-s001.zip › Supplementary Table S3. Descriptives of tissue gene expression data.pdf]

Supplementary Table S3. Descriptive statistics of tissue gene expression in different structures of the central nervous system.

CNS structure	Mean [range]; SD
Amygdala	-8.33e-008 [-1.81,1.64]; 1.00
Anterior cingulate cortex	1.08e-006 [-1.83, 1.40]; 1.00
Caudate nucleus	7.50e-007 [-0.22, 0.22]; 0.14
Cerebellum	8.33e-008 [-0.22, 0.32]; 0.14
Frontal cortex	-5.00e-007 [-2.83, 1.10]; 1.00
Hippocampus	-3.33e-007 [-0.30, 0.24]; 0.16
Hypothalamus	8.33e-008 [-2.16, 1.18]; 1.00
Nucleus accumbens	-1.67e-007 [-0.79, 0.35]; 0.33
Putamen	6.67e-007 [-0.60, 0.31]; 0.29
Spinal cord	-3.33e-007 [-0.25, 0.37]; 0.17
Substantia nigra	-7.50e-007 [-0.65, 0.37]; 0.31

Footnote: Mean, range (minimum, maximum), and standard deviation (SD) of normalized (z-scores) gene expression data are given per each central nervous system (CNS) structure. Values were calculated based on the means individual expression data of the 12 DCD-associated genes. Gene expression data were available as transcript per million.
