# Supplementary material for: Pathogenetic Insights into Developmental Coordination Disorder Reveal Substantial Overlap with Movement Disorders
Source: Brain Sci. 2023 Nov 23;13(12):1625. doi: 10.3390/brainsci13121625 (PMC10741651; doi:10.3390/brainsci13121625)
Supplement: Supplementary file 1 [file brainsci-13-01625-s001.zip › Supplementary Table S4. Predicted genes listed in movement disorder gene panels.pdf]

Supplementary Table S4. Association of DCD-predicted genes with movement disorders.

DCD-predicted gene	Movement disorder(s) which this gene is linked to
ATCAY	Early- and late-onset ataxia
ATP1A3	Early- and late-onset ataxia, dystonia
CACNA1A	Early- and late-onset myoclonus, early- and late-onset ataxia, dystonia, paroxysmal movement disorders
CHRNA4	Dystonia
EEF1A2	Myoclonus
KIF1A	Early-onset ataxia, spastic paraplegia
L1CAM	Spastic paraplegia
MAPT	Late-onset myoclonus
RTN2	Spastic paraplegia
SCN2A	Early- and late-onset myoclonus
SEMA6B	Early- and late-onset myoclonus
STXBP1	Early-onset ataxia, early- and late-onset myoclonus

Footnote. Table showing the association of 12 DCD-predicted genes with movement disorders. The 12 abovementioned genes are part of the 200 genes predicted to be functionally similar to the DCD-associated genes based on the network function of the program Metabrain (https://network.metabrain.nl). We compared these genes with genes known to be linked to movement disorders based on gene lists from our hospital (https://www.umcg.nl/-/afdeling/genetica/aanvragen-genoomdiagnostiek) as well as from the Task Force on Genetic Nomenclature in Movement Disorders (Lange LM, et al., Mov Disord. 2022;37(5):905-935). The 12 DCD-predicted genes were mainly associated with ataxia and myoclonus.
